# Effects of Tinnitus on Cognition and Depressive Symptoms Over Time

**DOI:** 10.1093/geroni/igab046.3485

**Published:** 2021-12-17

**Authors:** Jennifer De Anda, Teresa Warren, Tyler Bell, William Kremen, Carol Franz

**Affiliations:** 1 San Diego State University, San Diego, California, United States; 2 University of California San Diego, La Jolla, California, United States

## Abstract

Evidence suggests links among tinnitus, depression, and cognition. We examined these associations over time. We hypothesized baseline tinnitus would predict poorer cognitive performance and more depressive symptoms an average of 11.4 years later. We examined 839 men at two timepoints (baseline age M=55.94; follow-up age M=67.56). At each time point participants responded yes/no if they had tinnitus. We created three tinnitus status groups – no tinnitus at either time, tinnitus at both, and no tinnitus at baseline but tinnitus at follow-up. At both time points we measured cognitive performance with tests of episodic memory, processing speed, executive function, and verbal fluency. Depressive symptoms were based on the Center for Epidemiological Studies Depression scale. There was no association between tinnitus and any measure of cognitive performance. Depressive symptoms declined from baseline to follow-up. In separate mixed models predicting depressive symptoms, there was a significant main effect for tinnitus status at baseline (p = .003) and follow-up (p < .001). Those with tinnitus at both times had significantly higher depressive symptoms than the “No tinnitus” group (p < .001). This association remained significant after accounting for baseline depressive symptoms (p = .011) at follow-up. Results did not support the hypothesis that tinnitus would be associated with poorer cognitive function. However, depressive symptoms declined among those with no tinnitus than the other groups. The relationship between tinnitus and depressive symptoms may have implications for future cognitive performance among older adults, given previous evidence that depressive symptoms are risk factors for cognitive decline.

